# Impact of insight on treatment adherence in bipolar disorder

**DOI:** 10.1192/j.eurpsy.2025.1081

**Published:** 2025-08-26

**Authors:** F. Sahnoun, F. Cherif, D. Mnif, F. Guermazi, I. Baati, I. Feki, R. Masmoudi, J. Masmoudi

**Affiliations:** 1 Psychiatry A Department, Hedi Chaker University hospital, Sfax, Tunisia

## Abstract

**Introduction:**

Extreme mood swings are a hallmark of bipolar disorder (BD). Consistent treatment adherence, including medication and regular attendance at outpatient care, is usually necessary for the effective management of BD. However, many individuals with BD struggle with treatment engagement, which is often influenced by several factors, including insight.

**Objectives:**

This study aims to explore the relationship between patients’ level of insight into their condition and their attendance at scheduled treatments

**Methods:**

It was a cross-sectional, descriptive, and analytical study conducted on bipolar disorder patients from the Psychiatry “A” Department, Hedi Chaker University Hospital. Clinical and sociodemographic data were collected from March to September 2023 using a questionnaire along with Medication Adherence Rating Scale (MARS) for assessing treatment adherence and the Birchwood Insight Scale (BIS) to evaluate insight

**Results:**

A total of 37 patients with BD completed the questionnaire. The mean age was 45.4 ± 13.9 years, with a sex ratio (M/F) of 1.46.

In our study, 73% of patients were with BD type I and 27% were with BD type II.

The mean MARS score was 7.14 ± 2.13, and 37.8% of our population were non-adherent to medication.

The mean BIS score was 8.58 ± 2.35, and 56.8% of patients had good insight.

Mean scores of awareness of illness, re-labeling of symptoms, and need for treatment subscales of BIS were respectively 2.86, 2.73, and 2.86.

The MARS score was positively correlated with the BIS score (p = 0.002, r = 0.40).

The MARS score was also positively correlated with both the *awareness of illness* subscale of the BIS (p = 0.035, r = 0.34) and the *re-labeling of symptoms* subscale of the BIS (p = 0.41, r = 0.33).

**Conclusions:**

This study demonstrates that certain aspects of insight are significantly associated with treatment adherence in bipolar disorder. Specifically, a*wareness of illness* and *re-labeling of symptoms* suggest that the ability to reinterpret and understand symptoms may enhance engagement with care. In contrast, the *Need for Treatment* subscale did not show a significant correlation with attendance, indicating that simply recognizing the need for treatment alone may not be sufficient to ensure consistent participation in care.

**Disclosure of Interest:**

None Declared

